# Malnutrition and Related Factors in Older Patients With Gastrointestinal Cancer Receiving Chemotherapy

**DOI:** 10.7759/cureus.58252

**Published:** 2024-04-14

**Authors:** Diğdem Doğan Akagündüz, Hilal Şahin, Baran Akagündüz

**Affiliations:** 1 Nutrition and Dietetics, Mengücek Gazi Training and Research Hospital, Erzincan Binali Yıldırım University, Erzincan, TUR; 2 Nutrition and Dietetics, Faculty of Health Science, Erzincan Binali Yıldırım University, Erzincan, TUR; 3 Medical Oncology, Mengücek Gazi Training and Research Hospital, Erzincan Binali Yıldırım University, Erzincan, TUR

**Keywords:** gastrointestinal cancer, nutrition, malnutrition, cancer, older adult

## Abstract

Objectives

The incidence and mortality of gastrointestinal (GI) malignancies increase exponentially with age. Malnutrition is a documented poor prognostic factor in older patients with cancer. There is insufficient data about the prevalence of malnutrition and associated factors in older patients with GI cancer. Thus, we aimed to investigate the prevalence of malnutrition and related factors among older patients with GI cancer.

Methods

A total of 121 patients aged over 70 years diagnosed with various types of GI cancers applied to the medical oncology clinic included in this cross-sectional study. We evaluated the nutrition status with a mini-nutritional assessment (MNA) score.

Results

The prevalence of malnutrition was 76 (62.8%) in our study population. The mean age was 76.5 (range 70 to 90 years), and 71 (58.6%) were male. In the multivariate logistic regression model, lower BMI (OR: 3.379, 95% CI: 1.465-7.812, *p *= 0.005), having gastroesophageal cancer (OR: 5.797, 95% CI: 2.387-14.091, *p*<0.001), treating with palliative chemotherapy (OR: 4.597, 95% CI: 1.799-11.772, *p *= 0.002), and frailty according to G8 score (OR: 10.798, 95% CI: 4.495-25.924, *p*<0.001) were associated with malnutrition.

Conclusions

Our study revealed that palliative chemotherapy, low BMI, frailty, and gastroesophageal cancer are risk factors for malnutrition in older patients with GI cancer. Physicians need to be aware of patients who may be at risk for malnutrition. Patients at risk of malnutrition may benefit from interventions to enhance their nutrition. Further studies consisting of larger cohorts are needed to determine malnutrition and related factors in older patients with cancer.

## Introduction

Globally, gastrointestinal (GI) malignancies contribute to more than 30% of cancer incidence and 32% of cancer-related fatalities [1]. As age advances, the occurrence and mortality rates of GI malignancies rise significantly. Poor nutritional status is established as a detrimental prognostic indicator among elderly cancer patients, with malnutrition and heightened cancer susceptibility being more prevalent in older individuals [2]. Malnutrition is the main reason for 10% to 20% of cancer deaths, rather than cancer itself [3]. Malnutrition in older persons was described by the French National Authority for Health as one or more of the following: ≥5% loss of body weight in a month or ≥10% in six months, and/or a 17/30 mini-nutritional assessment (MNA) score, 35 g/L serum albumin, and/or a 21 kg/m2 BMI [4]. Older patients with cancer are more vulnerable due to the existence of comorbidities and geriatric syndromes such as malnutrition and depression, which negatively impact chemotherapy tolerance. Therefore, before making treatment decisions, older cancer patients should have a thorough geriatric assessment, with a focus on nutritional screening [5].

Several studies have been conducted to determine factors associated with malnutrition in patients with cancer who are older. A recent systematic review and meta-analysis identified the following as risk factors for malnutrition: eating disorders, dementia, signs of impaired swallowing efficacy, institutionalization, constipation, poor or moderate self-reported health status, frailty in hospitalized individuals, high rates of polypharmacy, general health decline, including physical function, and Parkinson's disease [6].

While malnutrition risk is frequent in older patients with cancer, it is significantly higher in individuals with GI malignancies, especially when GI symptoms such as anorexia, early satiety, nausea, vomiting, dysphagia, odynophagia, diarrhea, constipation, malabsorption, and pain are present [7]. Unintentional weight loss, primarily due to GI discomfort, occurs in certain patients well before cancer is diagnosed. In patients with locally advanced or metastatic GI cancers, weight loss at presentation has been linked to a lower ability to tolerate anti-cancer therapy, an increase in severe dose-limiting toxicities, a lower response rate, a worse quality of life, a decline in performance status, and a shorter survival outcome. Depending on the definition given in the literature and the kind of GI cancer, the prevalence varies from 28% to 54% for hepatocellular, 39% to 71% for colorectal, and 56% for pancreatic cancers [8]. In a study conducted on 313 GI cancer patients, the malnutrition rate was 52%. In this study among patients aged older than 70 years, 18% of the authors found that poor performance status, three or more lines of therapy, pancreatic cancer, and gastric cancer are factors associated with malnutrition [9]. However, there is insufficient data about the prevalence of malnutrition and associated factors in older patients with GI cancer. Thus, we aimed to determine the prevalence of malnutrition and affecting factors among older patients with GI cancer.

## Materials and methods

**Figure 1:**
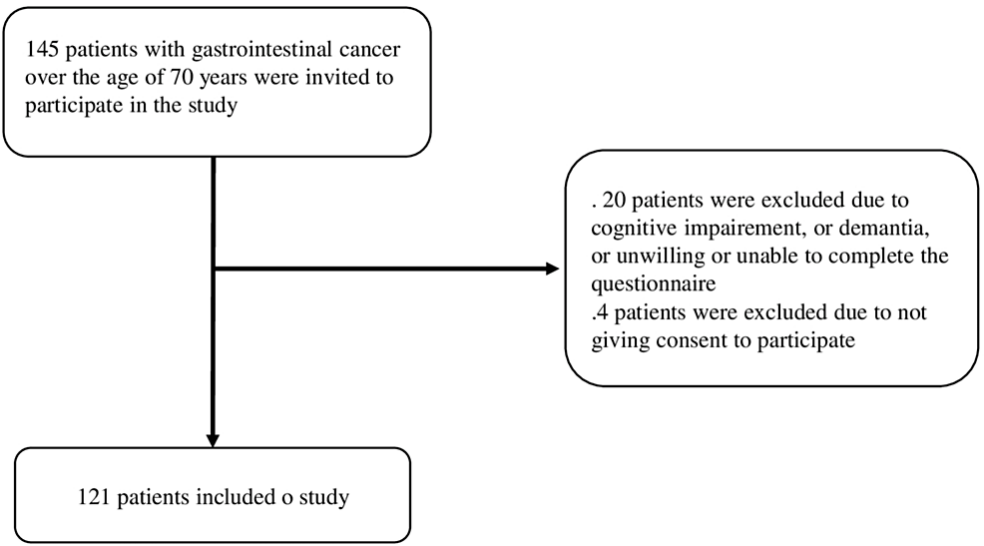
Flowchart of the study

A total of 121 patients aged over 70 years diagnosed with various types of GI cancers applied to the medical oncology clinic of Mengücek Gazi Research and Training Hospital in Erzincan, Turkey between February 15, 2021, and January 2, 2022, and were invited to participate in the present cross-sectional study. The study was approved by the ethics committee of the Mengücek Gazi Training and Research Hospital, Erzincan Binali Yıldırım University (approval no. E-88012460-050.04-337741). Patients diagnosed with non-GI cancer, younger than 70 years, instability due to acute cerebrovascular events, sepsis, acute renal injury, acute respiratory failure, patients with missing data, and patients with dementia or delirium were excluded from the study (Figure 1).

The demographic characteristics of the patient population, namely age, gender, marital status, education status, and history of falling, were collected by self-reporting. The cancer stage (local or advanced), cancer type, and chemotherapy type (adjuvant/neoadjuvant or palliative) were recorded from patient charts. Activities of daily living (ADLs) by Barthel et al. [10] and instrumental activities of daily living (IADL) by Lawton et al. [11] were the tools used for assessing the functional status of patients.

The 10 items that make up the Barthel score are: eating, dressing, toilet usage, chair/bed transfer, self-care, feeding, bathing, and using a ladder. Its scores fall between 0 and 100. Patients were classified as dependent if their score was lower than 90. The eight items on the Lawton scale are the ability to use the phone, shopping, cooking, cleaning, laundry, medication responsibility, mode of transportation, and financial management. Each item has a score of zero (low function, dependent, impairment) or one (high function, independent, no impairment). The original form did not measure these three items in males because cooking, cleaning, and laundry are among the tasks that are primarily performed by women. As a result, the overall score for men and women varied from zero to five and from zero to eight, respectively. An IADL score of less than 5 for men or less than 8 for women indicates dependency.

The Charlson comorbidity index (CCI) was used to evaluate comorbidity [12]. The cognitive state was assessed using the mini-mental state examination (MMSE) (a score of 24 to 26 considered mild cognitive impairment vs. ≥27, no cognitive impairment) [13]. The geriatric depression scale-15 (GDS-15) (≥5 is depressed) was used to determine psychological status [14]. According to the number of drugs recorded, the use of five or more drugs was defined as polypharmacy. Also noted were Eastern Cooperative Oncology Group-performance status (ECOG-PS) scores (classified as 0-1 vs. 2-4). The number of falls and fall-related injuries during the previous 12 months (presence or absence of falls) was used to gauge the patient's fall history. The BMI was calculated for each patient using the standardized formula: weight (kg)/height (m2). The G8 was used to detect frailty, and a score ≤14 was defined as frail in the patient population [15]. The MNA was performed on all patients to detect malnutrition risk. If the total score was 12-14, 8-11, or 0-7, it was accepted as normal nutrition, malnutrition risk, or malnutrition, respectively [16]. Patients with normal nutrition and malnutrition risk were defined as the non-malnutrition group.

For statistical analysis of the data, SPSS Statistics version 22 (IBM Corp., Armonk, NY, USA) was used. Qualitative variables were described by frequencies and percentages, continuous variables by mean and standard deviation, or median and range. The normal distribution range was determined by the Kolmogorov-Smirnov test. A T-test and a chi-square test were used to describe patient characteristics and compare subgroups of older patients with GI cancer with malnutrition and non-malnutrition. Variables that were shown to be significant by univariate analysis were subjected to logistic regression analysis. Regression analysis was utilized to generate the final model using the forward likelihood ratio (LR) test. Gender and age adjustments were made to the model, and variables with a p-value of less than 0.1 were included in the final version. The OR and 95% CI were shown as a consequence of the regression analysis. In all tests, results with p<0.05 were deemed statistically significant.

## Results

A total of 121 patients were included in the study. The prevalence of malnutrition was 76 (62.8%) in our study population. The mean age was 76.5 (range 70 to 90 years), and 71 (58.6%) were male. Age, female gender, diagnosis of gastroesophageal cancer, treatment with palliative chemotherapy, frailty, and low BMI were associated with malnutrition (Table 1). In the multivariate logistic regression model, low BMI (OR: 3.379, 95% CI: 1.465-7.812, p = 0.005), having gastroesophageal cancer (OR: 5.797, 95% CI: 2.387-14.091, p<0.001), treating with palliative chemotherapy (OR: 4.597, 95% CI: 1.799-11.772, p = 0.002), and frailty according to G8 score (OR: 10.798, 95% CI: 4.495-25.924, p<0.001) were associated with malnutrition (Table 2).

**Table 1:** Baseline characteristics according to malnutrition status (total n = 121) ADL: Activities of daily living, ECOG-PS: Eastern Cooperative Oncology Group-performance status, GDS: Geriatric depression scale, IADL: Instrumental activities of daily living, MMSE: Mini-mental state examination

Variables	Category	Total n:121 (%)	Non-malnutrition n: 45 (%)	Malnutrition n: 76 (%)	p-value
Age	Between 70 and 74 years	63 (52)	22 (48.8)	41 (53.9)	0.03
	Between 75 and 84 years	41 (33.8)	21 (46.6)	20 (26.3)	
	+85 years	17 (14.2)	2 (4.4)	15 (19.8)	
Gender	Male	71 (58.6)	31 (68.8)	40 (52.6)	0.02
	Female	50 (41.4)	14 (31.2)	36 (47.4)	
Cancer type	Colorectal	42 (34.7)	15 (33.4)	27 (35.6)	0.01
	Pancreatic	21 (17.3)	9 (20)	12 (15.7)	
	Hepatobiliary	14 (11.5)	10 (22.2)	4 (5.3)	
	Gastroesophageal	44 (36.3)	11 (24.4)	33 (43.4)	
Chemotherapy type	Adjuvant/neoadjuvant	66 (54.5)	20 (44.4)	46 (60.5)	<0.01
	Palliative	55 (45.5)	25 (55.6)	30 (39.5)	
Cancer Stage	II	40 (33)	16 (35.5)	24 (31.6)	0.35
	III	26 (21.5)	9 (8.9)	15 (19)	
	IV	55 (45.5)	25 (55.6)	30 (39.5)	
Frailty	Yes (G8 score≤14)	71 (58.6)	21 (46.6)	50 (65.7)	0.021
	No (G8 score >14)	50 (41.4)	24 (53.4)	26 (34.3)	
Comorbidity (Charlson comorbidity index)	0	62 (51.2)	22 (48.8)	40 (52.6)	0.67
	1	30 (24.7)	15 (33.3)	20 (26.3)	
	2	29 (23.9)	8 (17.7)	16 (21.1)	
Depression	Not depressed (GDS score<4)	76 (62.8)	27 (60)	49 (64.4)	0.13
	Depressed (GDS score≥4)	45(37.2)	18(40)	27(35.6)	
Polypharmacy	0-4 different drugs	67 (55.3)	25(55.5)	42(55.2)	0.98
	>5 drugs	54(44.7)	20(44.5)	34(44.8)	
ADL score	Dependent (score: <90)	56 (46.2)	19 (42.2)	37 (48.6)	0.71
	Independent (score ≥90)	65 (53.8)	26 (57.8)	39. (51.4)	
IADL score	Dependent: score <8 (female), <5 (male)	51 (42.1)	20 (44.5)	31 (40.7)	0.69
	Independent score 8 (female), 5 (male)	70 (57.9)	25 (55.5)	45 (59.3)	
ECOG-PS	0–1	89 (73.5)	30 (66.6)	59 (77.6)	0.82
	≥2	32 (26.5)	15 (33.3)	17 (22.4)	
BMI	Mean (SD)	25+/-1.2	26+/-1.4	24+/-1.1	0.04
MMSE score	Mean (SD)	22.1+/-1.9	21.1+/-1.7	22.6+/-1.6	0.93
Marital status	Single/widowed/divorced	91 (75.2)	36 (80)	67 (88.1)	0.55
	Married	30 (24.8)	9 (20)	9 (19.9)	
History of falling	Yes	15 (12.3)	6 (13.3)	9 (11.8)	0.12
	No	106 (87.7)	39 (86.7)	67 (88.2)	
Education status	<5 years	54 (46)	21 (42.2)	37 (48.6)	0.91
	≥5 years	65 (54)	26 (57.8)	39 (51.4)	

**Table 2:** Multivariate logistic regression model for malnutrition

Variables	OR	95% CI for OR		p-value
		Lower	Upper	
Age	987	0.895	1.063	0.296
Gender (female)	0.864	0.341	2.191	0.758
Cancer type (gastroesophageal)	5.797	2.387	14.091	<0.001
BMI	3.379	1.465	7.812	0.005
Chemotherapy type (palliative)	4.597	1.799	11.772	0.002
Frailty	10.798	4.495	25.924	<0.001

## Discussion

This study was conducted to evaluate malnutrition and related factors in older patients with GI cancer who received chemotherapy. Malnutrition is common in older patients with cancer, and it is related to survival and quality of life. Our study showed that the malnutrition rate was 62.8% in older patients with GI cancer treated with chemotherapy. Also, we determined that low BMI, diagnosis of gastroesophageal cancer, and treatment with palliative chemotherapy were factors associated with malnutrition in our population. We assessed nutritional status with MNA, which is validated in older patients with cancer. It is reported that 32% of cancer patients who applied to outpatient care using the Nutrition Risk Screening (NRS)-2002 were at risk of malnutrition [17]. Pressoir et al. demonstrated that older patients with GI cancer and head and neck cancer are at higher risk of malnutrition [18]. A recent retrospective study analyzing 3585 cancer patients showed that 28% of the cohort were at high risk of malnutrition. In this study, patients with upper and lower GI cancers had the highest prevalence of malnutrition [19]. A cross-sectional study demonstrated that malnutrition among older patients with cancer was 41.9% [20]. We found a higher rate of malnutrition compared to other studies. This may be attributed to the fact that our patient population consisted of older patients with GI cancer treated with chemotherapy.

There is a relationship between cancer stage and malnutrition. At the time of diagnosis, 15% to 20% of cancer patients have anorexia. Acute and chronic inflammation linked to the development of metastatic cancer contributes to the pathophysiology of malnutrition associated with cancer, even if anorexia is a prominent factor in malnutrition. In our study, the cancer stage did not affect malnutrition. This may be attributed to the small sample size and the heterogeneous nature of our study population. Low BMI is associated with poor nutritional status in cancer patients [21]. As predicted, we found low BMI to be associated with malnutrition in multivariate analysis. We found that, among GI cancers, gastroesophageal cancer was related to malnutrition. This may be attributed to high symptom burdens like dysphagia, odynophagia, nausea, and vomiting.

In this analysis, we showed that treatment with palliative chemotherapy is related to malnutrition. Malnutrition may result from both acute and long-term complications of cancer therapies. A good diet increases the likelihood that older cancer patients will be able to withstand their treatment's negative effects. They need enough calories and protein to stay strong and maintain healthy tissues. Patients have different adverse effects according to their particular malignancy, therapy type, duration, and dosage.

The nutritional status of older individuals with cancer can be significantly impacted by the disease and the treatment. Chemotherapy and radiation therapy are anticancer treatments that can cause nausea, asthenia, exhaustion, and vomiting. Malnutrition, on the other hand, may affect the effectiveness of cancer surgery, radiation, and chemotherapy by changing the dynamics of healing, patient metabolism, and pharmacokinetics. On the other side, malnutrition can alter the pharmacokinetics, healing dynamics, and patient metabolism, which can impact the effects of cancer surgery, radiation, and chemotherapy [22]. Research indicates that in elderly individuals undergoing chemotherapy, a low MNA score was found to be independently linked to non-hematologic damage [23]. Reduced BMI, sarcopenia, and weight loss have all been linked to higher treatment toxicity [24].

Frailty is a geriatric syndrome described as “decreased physiological capabilities and reserve to maintain homeostasis after a stressor” [25]. Frailty is accounted for in about 43% of older patients with cancer [26]. It is a poor prognostic factor for older patients with cancer. One of the most frequently used scales for frailty is the Fried frailty index, which consists of self-reported exhaustion, slow walking speed, low physical activity, reduced handgrip strength, and unintentional weight loss [25]. One of the most important components required for the definition of both malnutrition and frailty is weight loss. Frailty and malnutrition occur together in many elderly individuals. The other shared determinant includes sociodemographic, physical, and cognitive correlates. Weight loss is a common side effect of cancer therapy for older individuals, as well as other age-related illnesses. Furthermore, compared to non-frail patients, frail patients are more likely to suffer from cognitive problems, polypharmacy, reduced mobility, poor eating dependence, and anorexia, all of which are linked to an increased risk of malnutrition [27]. Numerous research investigations have documented the correlation between undernourishment and fragility. According to a recent study, there is an eight-fold higher chance of frailty in those who are malnourished, indicating that malnutrition is an independent predictor of frailty [28]. A substantial correlation between malnutrition and frailty was also found in another study [29]. It is unclear, therefore, if malnutrition causes frailty or if frailty causes malnutrition. Additional investigation is required to assess the correlation. We used G8 to detect frailty, with a score of 14 as the cutoff.

The limitations of this study must be taken into consideration when interpreting the results. Our study population was heterogeneous, consisting of different types of GI cancers. Moreover, patients were at different stages of cancer and receiving different chemotherapy regimens. This is a single-center study, and the study population was relatively small. Finally, we did not perform a full, comprehensive geriatric assessment of our patients.

## Conclusions

In this study, we found that malnutrition is common in older patients with GI cancer. Chemotherapy remains the backbone of treatment. We observed that frailty, palliative chemotherapy, low BMI, frailty, and gastroesophageal cancer are risk factors for malnutrition in older patients with GI cancer receiving chemotherapy. Physicians need to be aware of patients who may be at risk for malnutrition. Patients at risk of malnutrition may benefit from interventions to enhance their nutrition. Further studies consisting of larger cohorts are needed to determine malnutrition and related factors in older patients with cancer. Methods with high specificity and sensitivity should be developed to more accurately detect malnutrition in older patients with cancer.
